# Discovery and functional characterization of *N*-(thiazol-2-yl)-benzamide analogs as the first class of selective antagonists of the Zinc-Activated Channel (ZAC)

**DOI:** 10.1016/j.bcp.2021.114782

**Published:** 2021-09-21

**Authors:** Nawid Madjroh, Eleni Mellou, Paul A. Davies, Pella C. Söderhielm, Anders A. Jensen

**Affiliations:** a Department of Drug Design and Pharmacology, Faculty of Health and Medical Sciences, University of Copenhagen, 2100 Copenhagen Ø, Denmark; b Department of Neuroscience, Tufts University School of Medicine, Boston, MA, USA

**Keywords:** Cys-loop receptor (CLR), Pentameric ligand-gated ion channel (pLGIC), Zinc-Activated Channel (ZAC), *N*-(thiazol-2-yl)-benzamide analogs, Negative allosteric modulator (NAM), State-dependent inhibition

## Abstract

The Zinc-Activated Channel (ZAC) is an atypical member of the Cys-loop receptor (CLR) superfamily of pentameric ligand-gated ion channels, with its very different endogenous agonists and signalling properties. In this study, a compound library screening at ZAC resulted in the identification of 2-(5-bromo-2-chlorobenzamido)-4-methylthiazole-5-methyl ester (**1**) as a novel ZAC antagonist. The structural determinants for ZAC activity in **1** were investigated by functional characterization of 61 analogs at ZAC expressed in *Xenopus* oocytes by two-electrode voltage clamp electrophysiology, and couple of analogs exerting more potent ZAC inhibition than **1** were identified (IC_50_ values: 1–3 μM). **1** and *N*-(4-(tert-butyl)thiazol-2-yl)-3-fluorobenzamide (5**a**, TTFB) were next applied in studies of the functional properties and the mode of action of this novel class of ZAC antagonists. TTFB was a roughly equipotent antagonist of Zn^+^- and H^+^ -evoked ZAC signaling and of spontaneous ZAC activity, and the slow on-set of its channel block suggested that its ZAC inhibition is state-dependent. TTFB was found to be a selective ZAC antagonist, exhibiting no significant agonist, antagonist or modulatory activity at 5-HT_3_A, α_3_β_4_ nicotinic acetylcholine, α_1_β_2_γ_2s_ GABA_A_ or α_1_ glycine receptors at 30 μM. **1** displayed largely noncompetitive antagonism of Zn^2+^-induced ZAC signalling, and TTFB was demonstrated to target the transmembrane and/or intracellular domains of the receptor, which collectively suggests that the *N*-(thiazol-2-yl)-benzamide analog acts a negative allosteric modulator of ZAC. We propose that this first class of selective ZAC antagonists could constitute useful pharmacological tools in future explorations of the presently poorly elucidated physiological functions governed by this CLR.

## 1. Introduction

The pentameric ligand-gated ion channels in the Cys-loop receptor (CLR) superfamily are mediators of the fast signalling mediated by the classical neurotransmitters acetylcholine (ACh), serotonin (5-hydroxy-tryptamine, 5-HT), γ-aminobutyric acid (GABA) and glycine [1], The nicotinic ACh, GABA_A_, 5-HT_3_ and glycine receptors (nAChRs, GABA_A_Rs, 5-HT_3_Rs and GlyRs, respectively) exert and regulate a plethora of physiological functions in the central nervous system and in the periphery, and the receptors are targeted by drugs currently used to treat insomnia/sleep disorders, anxiety, epilepsy, nicotine addiction, muscle spasms and nausea/emesis and are being pursued as putative therapeutic targets for numerous other indications [2–8]. The membrane-embedded pentameric CLR complex is composed of an extracellular domain (ECD) made up by the *N*-termini of the five subunits, a transmembrane domain (TMD) consisting of the four membrane-spanning α-helices from each of the subunits, and an intracellular domain (ICD) made up by the second intracellular loops of the five subunits [9–13]. Signal transduction through the classical CLR is initiated by agonist binding to orthosteric sites in subunit interfaces in the ECD, which triggers opening of and ion flux through the ion channel in the transmembrane domain (TMD) until the receptor undergoes deactivation or desensitization [12–14]. These conformational transitions of the CLR are highly sensitive to ligand binding to allosteric sites in the pentameric complex, and positive and negative allosteric modulators (PAMs and NAMs, respectively) figure prominently as pharmacological tools and therapeutics for the classical CLRs [4,15–20].

The Zinc-Activated Channel (ZAC) is in many ways an atypical CLR [21,22]. ZAC constitutes the fifth branch of the phylogenetic tree over mammalian CLRs and exhibits low amino acid sequence homology with other CLR subunits [21]. When expressed in HEK293 and COS-7 cells, ZAC forms homomeric cation-selective channels activated by zinc (Zn^2+^), copper (Cu^2+^) and protons (H^+^), with the divalent cations Ca^2+^ and Mg^2+^ inhibiting channel signalling [21,22]. In addition to the vastly different sizes and physico-chemical properties of the identified ZAC agonists compared to the classical CLR neurotransmitters, the signaling properties of the recombinant ZAC are also very distinct from those displayed by the classical CLRs, with ZAC exhibiting considerable levels of spontaneous activity and very slow desensitization kinetics in patch-clamp recordings from HEK293 and COS-7 cells [21,22]. Albeit an atypical CLR, the existence of a ligand-gated ion channel acting as an *in vivo* sensor of fluctuations in endogenous zinc, copper and/or pH levels does not seem unlikely. However, in contrast to the detailed insight into *in vivo* functions of the classical CLRs obtained through decades of research, very little is known about the physiological roles governed by ZAC. Whereas the human *ZACN* gene is conserved in most other mammalian species, orthologous genes are absent from the rat and mouse genomes, which in part explains the few studies of the physiological functions of the receptor performed to date. ZAC mRNA has been detected in fetal and adult human brain and in pancreas, placenta, prostate, thyroid, trachea and stomach tissues [21,23]. Recently, ZAC has been proposed to be expressed in thymus and lymph organs and to be important for T-cell division [24], but presently data to support this or any other claims for physiological functions of the receptor are very sparse.

Explorations into the *in vivo* functions governed by ZAC and the therapeutic potential in the receptor would be facilitated by the availability of potent and selective pharmacological tools. The curare alkaloid tubocurarine (TC) has been shown to inhibit ZAC signalling with low-micromolar IC_50_ values [21,25], but the fact that TC is a promiscuous antagonist displaying comparable or even higher antagonist potencies at several other CLRs makes it unsuited as a pharmacological tool for ZAC [26,27]. A recent patent application has reported that a wide range of structurally diverse ligands (including ATP, dye compounds, heparin and tricyclic antidepressants) modulate ZAC signalling at mid-micromolar to millimolar concentration ranges [24]. While this suggests that ZAC analogously to other CLRs is susceptible to allosteric modulation, the moderately potent ZAC modulation and the potent off-target activities that characterize all of these compounds also render them inapplicable as pharmacological tools [24]. In the present work, we have attempted to remedy this lack of pharmacological tools by a search for novel modulators of ZAC based on a screening of a small compound library at the receptor. We report the discovery of the first class of selective ZAC antagonists and present the functional properties of and the mode of action for their receptor inhibition.

## 2. Materials and methods

### 2.1. Materials

Z_n_Cl_2_, CuCl_2_, 5-HT, GABA, (*S*)-nicotine, glycine and all chemicals for buffers were purchased from Sigma-Aldrich (St. Louis, MO), and culture medium, serum, antibiotics and trypsin for cell culture were obtained from Invitrogen (Paisley, UK). The 1,680 compound-library (the same library as applied in a previous study [28]) was purchased from Chembridge Corporation (San Diego, CA), and analogs of the identified screening hit (compound **1**) were obtained from Chembridge Corporation, Enamine (Kiev, Ukraine) and ChemDiv (San Diego, CA). The PolyFect transfection reagent and the FLIPR Membrane Potential Red (FMP) assay kit were obtained from Qiagen (Hilden, Germany) and Molecular Devices (Crawley, UK), respectively. Defolliculated stage V-VI oocytes harvested from female *Xenopus laevis* frogs were obtained from Lohmann Research Equipment (Castrop-Rauxel, Germany). The construction of cDNAs for wild-type (WT) human ZAC (ZAC^Thl128^ [25]) and other CLR subunits (ZAC-pCIneo, ZAC-pUNIV, m5-HT3A-pUNIV, hαl-GlyR-pUNIV, hαl -GABA_A_R-pCDNA3.1 hβ2-GABA_A_R-pCDNA3.1, hγ2S-GABA_A_R-pCDNA3.1, hα3-nAChR-pCDNA3.1 and hβ4-nAChR-pCDNA3.1) and for chimeric subunits (m5-HT_3_A/ZAC-pUNIV and ZAC/hα_1_-Gly-pUNIV) have been described previously (the prefixes “h”, “m” and “r” denote himian, mouse and rat, respectively) [25,29–31]. The HEK293, h5-HT_3_A-HEK293 and rα_3_β_4_-nAChR-HEK293 cell lines were generous gifts from Drs. Jakob L. Hansen, Jan Egebjerg and Ken J. Kellar, respectively, and the construction of the stable HEK293 cell line expressing the human excitatory amino acid transporter subtype 3 (hEAAT3) has been described previously [32].

### 2.2. Cell culture and construction of the ZAC-HEK293 cell line

HEK293 cells were propagated in culture medium [Dulbecco’s Modified Eagle Medium Glutamax^™^-I supplemented with penicillin (100 U/mL), streptomycin (100 μg/mL) and 5% dialyzed fetal bovine serum] in a humidified atmosphere at 37 °C and 5% CO_2_. The ZAC-HEK293, h5-HT_3_A-HEK293 and rα_3_β_4_-nAChR-HEK293, and hEAAT3-HEK293 cell lines were grown in the same culture medium supplemented with 1 mg/mL G-418. For the construction of the stable monoclonal ZAC-HEK293 cell line, 5 × 10^5^ HEK293 cells were seeded in a 10 cm tissue culture dish, and 24 h later the cells were transfected with a total of 8 μg ZAC-pCIneo using PolyFect. 16 h after, the transfection medium were replaced with culture medium supplemented with 2 mg/mL G-418, and the cells were propagated in this medium for the following 3–4 weeks. Antibiotic-resistant individual cell clones were isolated and amplified in the continued presence of 1 mg/mL G-418. The individual clones were screened for functionality in the FMP assay (section 2.3).

### 2.3. FLIPR^®^ Membrane Potential Red (FMP) assay

The screening of the 1,680 compounds in the compound library for antagonist activity at the ZAC-HEK293 cell line was performed in the FMP assay essentially as previously described [33]. The day before the assay, cells were split into poly-D-lysine-coated black 96-well plates with clear bottoms (BD Biosciences, Palo Alto, CA) (6 × 10^4^ cells/well). The following day, the culture medium was aspirated and the wells were washed once with 100 μl assay buffer (140 mM NaCl, 4.7 mM KCl, 2.5 mM CaCl_2_, 1.2 mM MgCl_2_, 11 mM HEPES, 10 mM D-glucose, pH 7.4), after which 100 μl assay buffer supplemented with FMP dye (0.5 mg/ml) and the various test compounds were added to the wells. The plate was then incubated at 37 °C for 30 min and assayed in a NOVOStar plate reader (BMG Labtechnologies, Offenburg, Germany) measuring emission at 560 nm caused by excitation at 530 nm before and up to 1 min after the addition of 33 μl of agonist solution. In the screening, the 1,680 compounds in the compound library were tested at assay concentrations in the 10–30 μM range, using Cu^2+^ as agonist at an assay concentration of 200 μM.

### 2.4. Xenopus oocytes and two-electrode voltage clamp (TEVC) recordings

The cDNAs for the various WT and chimeric CLR subunits were linearized and subsequently transcribed and capped mMessage mMachine T7 RNA transcription kit (Ambion, Waltham, MA). Different amounts of cRNA mixtures were injected for ZAC (9.2–36.8 nL in a concentration of 0.05 μg/μL), 1ια_3_β_4_ nAChR (9.2–36.8 nL in a concentration of 0.1 μg/μL of each subunit in a ratio of 1:1), hα_1_ GlyR (9.2–36.8 nL in a concentration of 0.05 μg/μL), hα_1_β_2_γ_2s_ GABA_A_R (9.2–36.8 nL in a concentration of 0.05 μg/μL of each subunit in a ratio of 1:1:3), m5-HT_3_AR (9.2–36.8 nL in a concentration of 0.05 μg/μL), m5-HT_3_A/ZAC (9.2–36.8 nL in a concentration of 0.207 μg/μL), and ZAC/hai-Gly (9.2–36.8 nL in a concentration of 0.206 μg/μL). Oocytes were incubated in a sterile modified Barth’s solution [88 mM NaCl, 1 mM KCl, 15 mM HEPES (pH 7.5), 2.4 mM NaHCO_3_, 0.41 mM CaCl_2_, 0.82 mM MgSO_4_, 0.3 mM Ca(NO_3_)_2_, 100 U/ml penicillin and 100 μg/ml strep-tomycin] at 18 °C for 2 days after injection.

On the day of experiment, all compound dilutions were prepared in a saline solution [115 mM NaCl, 2.5 mM KCl, 10 mM MOPS (pH 7.5), 1.8 mM CaCl_2_, 0.1 mM MgCl_2_] and pH adjusted to 7.5 (if needed). Oocytes were placed in a recording chamber continuously perfused with this saline solution, and the compounds were applied in the perfusate. Both voltage and current electrodes were agar-plugged with 3 M KCl with a resistance of 0.2–2.0 MΩ. Oocytes were voltage-clamped at −50 mV (unless otherwise stated) by a Gene Clamp 500B amplifier (Axon Instruments, Union City, CA), while current signals were digitized by a Digidata 1320. Currents were recorded using pCLAMP 10 (Molecular Devices, Sunnyvale, CA). The recordings were performed at room temperature.

In all recordings, the compounds or compound combinations were applied in the bath until the peak current decayed to a steady state (up to 30 sec at the most). At the beginning or end (wherever appropriate) of all recordings determining concentration-response relationships at ZAC, two consecutive applications of a Zn^2+^ concentration giving rise to a maximal Zn^2+^ current (Zn^2+^ I_max_) were applied to the perfusate, and it was verified that these consecutive applications elicited responses of comparable current amplitudes (±20%). The functional properties of the modulators at the CLRs were determined by pre-application of the modulator to the perfusate 30 sec and followed by co-application of the modulator and the agonist to the perfusate. In all recordings, washes of 1–5 min were executed between the applications to prevent receptor desensitization, the duration of these washes depending on the kinetics of the respective receptors and the “stickyness” of the respective compounds. For determination of the concentration-inhibtion relationships exhibited by the modulators at ZAC, agonist concentrations around EC_50_ (1 mM Zn^2+^ and 1 μM H^+^/pH 6.0) were used. For the selectivity and mode-of-action studies, the agonist concentrations used for different CLRs or chimeric CLRs were in the EC_20_–EC_40_ range (verified on the day of experiment), as this enabled us to investigate both for putative positive and negative receptor modulation exerted by TTFB (hα_3_β_4_ nAChR, 3 μM (*S*)-nicotine; m5-HT_3_AR, 2 μM 5-HT; hα_1_ GlyR, 100 μM glycine; hα_1_β_2_γ_2S_ GABA_A_R, 30 μM GABA; m5-HT_3_A/ZAC, 0.3 μM 5-HT; ZAC/hɑ_1_-Gly, 2 μM Zn^2+^).

### 2.5. Data analysis

Data from the FMP assay was analysed using KaleidaGraph 3.08 (Synergy Software, Reading, PA). Concentration-response curves for Cu^2+^ and concentration-inhibition curves for compound **1** were constructed based on the difference in the relative fluorescence units (RFU) between the maximal fluorescence recorded before and after application of Cu^2+^. The data were fitted to sigmoidal curves with variable slopes using nonlinear regression, and EC_50_ (Cu^2+^) and IC_50_ (compound **1**) values were derived from these equations.

Data analysis of the results from the TEVC recordings were performed using Clampfit software version 10.5 (Molecular Devices, Crawley, UK) and GraphPad Prism version 7.0c (GraphPad Software, Inc. La Jolla, CA). Data for the test compounds were normalized to either the maximal agonist-evoked current or to an EC_20_–EC_50_-mediated response elicited by the agonist on each oocyte (specified in each case). Unless otherwise stated, data for agonist-induced responses in the presence of the antagonists were extracted from the plateau (or steady-state) of the agonist-evoked current. Concentration-response and concentration-inhibition curves were fitted in GraphPad Prism by nonlinear regression using the equation for sigmoidal dose-response with variable slope. I-V relationships were analyzed by fitting the curves with a third order polynomial model. Each data point represents the mean ± S.E.M. value of recordings performed on at least five oocytes from at least two different batches.

## 3. Results

### 3.1. Discovery of a novel ZAC antagonist

**Fig. 1.**
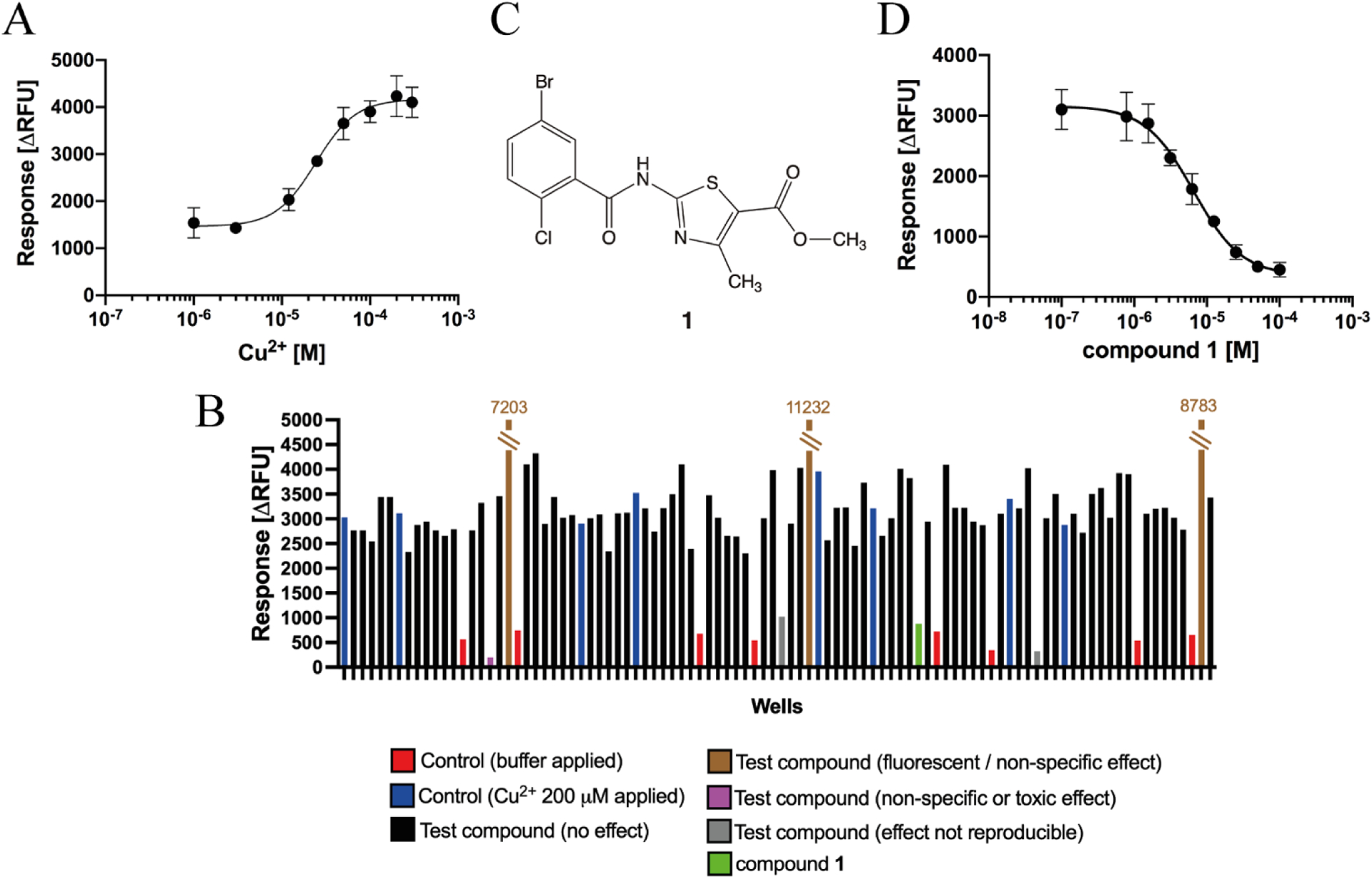
Identification of a novel ZAC antagonist. **A**. Concentration-response curve for Cu^2+^ at ZAC-HEK293 cells in the FMP assay. Data are from a single representative experiment performed in duplicate (of a total of 4) and are given as ΔRFU (mean ± S.D.) values. **B**. Data obtained for one 96-well plate (out of of a total of 21 96-well plates) tested in the screening at the ZAC-HEK293 cell line in the FMP assay using Cu^2+^ (200 μM) as agonist, more spefically the 96-well plate containing compound **1**. The data for the 16 wells in the 96-well plate containing controls [where the cells in the absence of any test compound were challenged with either pure buffer (*red*) or buffer supplemented with Cu^2+^ (200 μM) (*blue*)] and for the 80 wells in the plate containing various test compounds that either displayed negligible effects on the Cu^2+^ (200 μM)-induced response (*black*), were fluorescent or mediated non-specific effects *(brown)*, mediated non-specific or toxic effects *(purple)*, exhibited significant inhibition that not be reproduced in subsequent experiments (*grey*), and compound **1** *(green)* are indicated. **C**. Chemical structure of compound **1. D**. Concentration-inhibition curve for compound **1** at ZAC-HEK293 cells in the FMP assay using Cu^2+^ (200 μM) as agonist. Data are from a single representative experiment performed in duplicate (of a total of 3) and are given as ΔRFU (mean ± S.D.) values.

In search for novel ZAC modulators we developed a stable ZAC-HEK293 cell line to enable screening of a compound library at the receptor. ZAC-HEK293 clones were screened for functionality in the FMP assay by testing the ability of Cu^2+^ (200 μM) to elicit a significant increase in fluorescence levels (arising from the depolarization of the cells caused by ZAC activation). In the clone eventually used for the screening, Cu^2+^ consistently mediated concentration-dependent responses and displayed an average EC_50_ value of 26 μM (PEC_50_ ± S.E.M.: 4.59 ± 0.17, n = 4) in the FMP assay (Fig. 1A). Thus, Cu^2+^ exhibited a ~6-fold lower agonist potency in this assay than that determined for the metal ion at ZAC-expressing COS-7 cells in patch-clamp recordings [22]. Importantly, however, application of Cu^2+^ at concentrations up to 300 μM did not evoke any responses in HEK293 or h5-HT_3_A-HEK293 cells in the FMP assay (data not shown), indicating that the Cu^2+^-induced response in the ZAC-HEK293 cells was indeed mediated through the receptor. On this basis we decided to perform the compound library screening.

The 1,680 compounds in the compound library were screened for antagonist activity (at assay concentrations in the 10–30 μM range) at the ZAC-HEK293 cells in the FMP assay using Cu^2+^ (200 μM) as agonist (~8-fold higher concentration than Cu^2+^ EC_50_, Fig. 1A). Several compounds from the library were found to mediate substantial reductions in the Cu^2+^-evoked fluorescence response in the ZAC-HEK293 cells in the assay (Fig. 1B). However, the majority of these hits were ruled out as ZAC antagonists in subsequent experiments, where the apparent ZAC inhibition exhibited by some of the compounds in the screening could not be reproduced, while other screening hits were found to also reduce the glutamate-induced response in hEAAT3-HEK293 cells in the FMP assay, suggesting that their effects were either non-specific or due to cell toxicity (data not shown). Nevertheless, the screening resulted in the identification of one verifiable ZAC antagonist: 2-(5-bromo-2-chlor-obenzamido)-4-methylthiazole-5-methyl ester (compound **1**, Fig. 1B and 1C). Compound **1** was found to mediate concentration-dependent inhibition of the Cu^2+^ (200 μM)-evoked response in the ZAC-HEK293 cells in the FMP assay, exhibiting an IC_50_ value of 6.1 μM (pIC5o ± S. E.M.: 5.21 ± 0.13, n = 3) (Fig. 1D). Moreover, compound **1** did not affect the glutamate-induced response in hEAAT3-HEK293 cells, the 5-HT-induced response in h5-HT_3_A-HEK293 cells or the (*S*)-nicotine-induced response in rα_3_β_4_-nAChR-HEK293 cells in the FMP assay significantly at concentrations up to 100 μM (data not shown). Thus, **1** seemed to exert its effects on the Cu^2+^-evoked response in ZAC-HEK293 cells in the FMP assay through the receptor.

### 3.2. Functional properties of compound 1 at ZAC in Xenopus oocytes

**Fig. 2.**
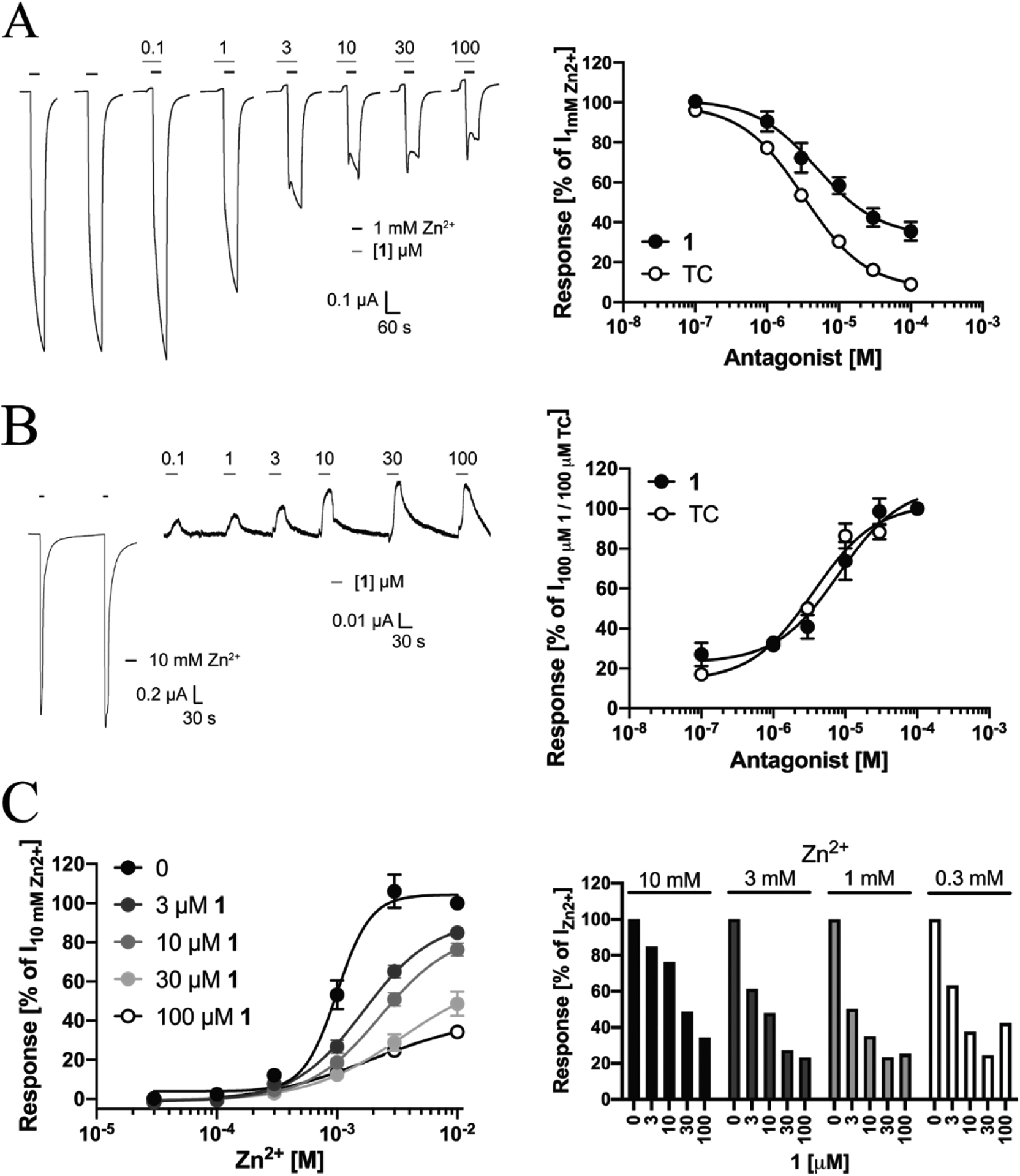
Antagonist properties displayed by compound 1 at ZAC expressed in Xenopus oocytes in TEVC recordings. **A**. Compound **1** inhibits Zn^2+^ -evoked currents through ZAC in oocytes. Representative traces of Zn^2+^ (1 mM) evoked currents in ZAC-expressing oocytes in the absence and in the presence of increasing concentrations of **1** *(left)*, and averaged concentration-inhibition relationships for **1** and TC at Zn^2+^ (1 mM)-induced currents in the oocytes *(right)*. Data for **1** are given as mean ± S.E.M. values (n = 6), and the tubocurarine (TC) data given for comparison are from a recent study [25], **B**. Compound **1** inhibits the spontaneous ZAC activity in oocytes. Representative traces of the effects of increasing concentrations of **1** on the leak current in ZAC-expressing oocytes (*left*), and averaged concentration-inhibition relationships for **1** and TC at tire spontaneous currents of ZAC in the oocytes *(right)*. Data for **1** are given as mean ± S.E.M. values (n = 6), and the tubocurarine (TC) data given here for comparison are from a recent study [25]. **C**. Delineation of the mode of antagonism exerted by compound **1** at Zn^2+^ -evoked ZAC currents in oocytes. *Left:* Averaged concentration–response relationships displayed by Zn^2+^ at ZAC in tire absence and in the presence of various concentrations of **1**. Data are given as mean ± S.E.M. values in % of the current evoked by 10 mM Zn^2+^ in the absence of **1** (I_10 mM Zn2+_) in the specific oocyte (n = 5–8). *Right:* The relative degrees of inhibition mediated by different concentration of **1** of the responses evoked by 10 mM, 3 mM, 1 mM and 0.3 mM Zn^2+^ through ZAC. Data (extracted from the data in Fig. 2C, *left*) are given as mean values in % of the current evoked by the specific Zn^2+^ concentration in the absence of **1** (I_Zn2+_).

The antagonism displayed by **1** at the ZAC-HEK293 cells in the FMP assay was subsequently verified at ZAC expressed in *Xenopus* oocytes in TEVC recordings. The signalling properties exhibited by ZAC in this assay has been characterized in detail in a previous study [25], and insights gained from that study guided the design of these experiments. 1 was found to inhibit the currents evoked by 1 mM Zn^2+^ (~EC_50_) through ZAC-expressing oocytes in a concentration-dependent manner. Interestingly, **1** appeared to mediate partial inhibition of the receptor, since the fitted concentration-inhibition curve for the compound plateaued at a response level constituting ~31% of that evoked by Zn^2+^ (1 mM) on its own, with **1** displaying an IC_50_ of 4.1 μM (pIC_50_ ± S.E.M: 5.39 ±0.17, n = 6) within this −69% inhibition range (Fig. 2A). Even though **1** thus seemingly displayed partial antagonist activity at ZAC, the comparable inhibition exerted by 30 μM and 100 μM **1** and the resulting plateau of the concentration-inhibition curve could also be a reflection of limited solubility of the compound at high concentrations, and it should be noted that several close structural analogs to **1** all displayed full antagonism at ZAC (see section 3.3.4). In view of this, we find it unlikely that **1** is a true partial antagonist of the receptor.

Analogously to the robust spontaneous activity exhibited by ZAC in mammalian cells [21,22], the channel also displays this characteristic when expressed in oocytes, albeit to a smaller degree [25], As can be seen from the traces in Fig. 2A, the 30 s preincubation of the ZAC-expressing oocyte with compound **1** prior to the co-application of **1** and Zn^2+^ onto the oocyte produced small but significant outward currents, reflecting a block of this spontaneous activity (Fig. 2A). When the direct effects of compound **1** at this spontaneous activity in ZAC-expressing oocytes subsequently were investigated in more detail, the antagonist was found to exhibit an IC_50_ value of 7.4 μM (PIC_50_ ± S.E.M: 5.13 ± 0.07, n = 8) at the receptor (Fig. 2B). The antagonist properties exhibited by TC at ZAC-expressing oocytes in parallel recordings are given in Fig. 2A and 2B for comparison (data are from a recent study [25]). In contrast to 1, TC mediated complete inhibition of the Zn^2+^ (1 mM)-induced response through ZAC (a fitted ~94% inhibition range), but with an IC_50_ of 3.2 μM (pIC_50_ ± S.E.M: 5.49 ± 0.04, n = 8) TC was equipotent with **1** as a ZAC antagonist (Fig. 2A) [25]. TC was also equipotent to **1** in its block of the spontaneous activity in ZAC-expressing oocytes with an IC_50_ of 3.4 μM (PIC_50_ ± S.E.M: 5.47 ± 0.04, n = 4) (Fig. 2B).

The antagonist properties of **1** at ZAC were next characterized in further detail by determining the concentration-response relationships for Zn^2+^ at the receptor in the absence and presence of various concentrations of the compound (Fig. 2C). Since several of the fitted concentration-response curves for Zn^2+^ obtained in presence of **1** did not reach convincingly saturation within the concentration ranges tested, it was not possible to extract reliable agonist EC_50_ values from the fitted curves, but visual inspection of the curves suggests that the presence of **1** in the assay reduces the agonist potency of Zn^2+^ at ZAC somewhat (Fig. 2C, *left*). However, much more pronounced than this effect on Zn^2+^ potency is the concentration-dependent **1**-mediated suppression of the maximal current evoked by Zn^2+^ through ZAC, which is indicative of a non-competitive antagonism mode (Fig. 2C, *left*). In further support of this, extraction and depiction of the data (from Fig. 2C, *left*) in a different manner revealed that **1** displays very similar concentration-inhibition relationships at the currents evoked by 0.3 mM, 1 mM and 3 mM Zn^2+^ through ZAC, whereas the antagonist appears less potent at the Zn^2+^ (10 mM)-evoked current through the receptor (Fig. 2C, *right*). All in all, the antagonism exerted by **1** at ZAC thus appeared to be largely non-competitive in its nature.

### 3.3. Stnicture-activity relationship (SAR) of the *N*-(thiazol-2-yl)-benzamide analog at ZAC

**Fig. 3.**
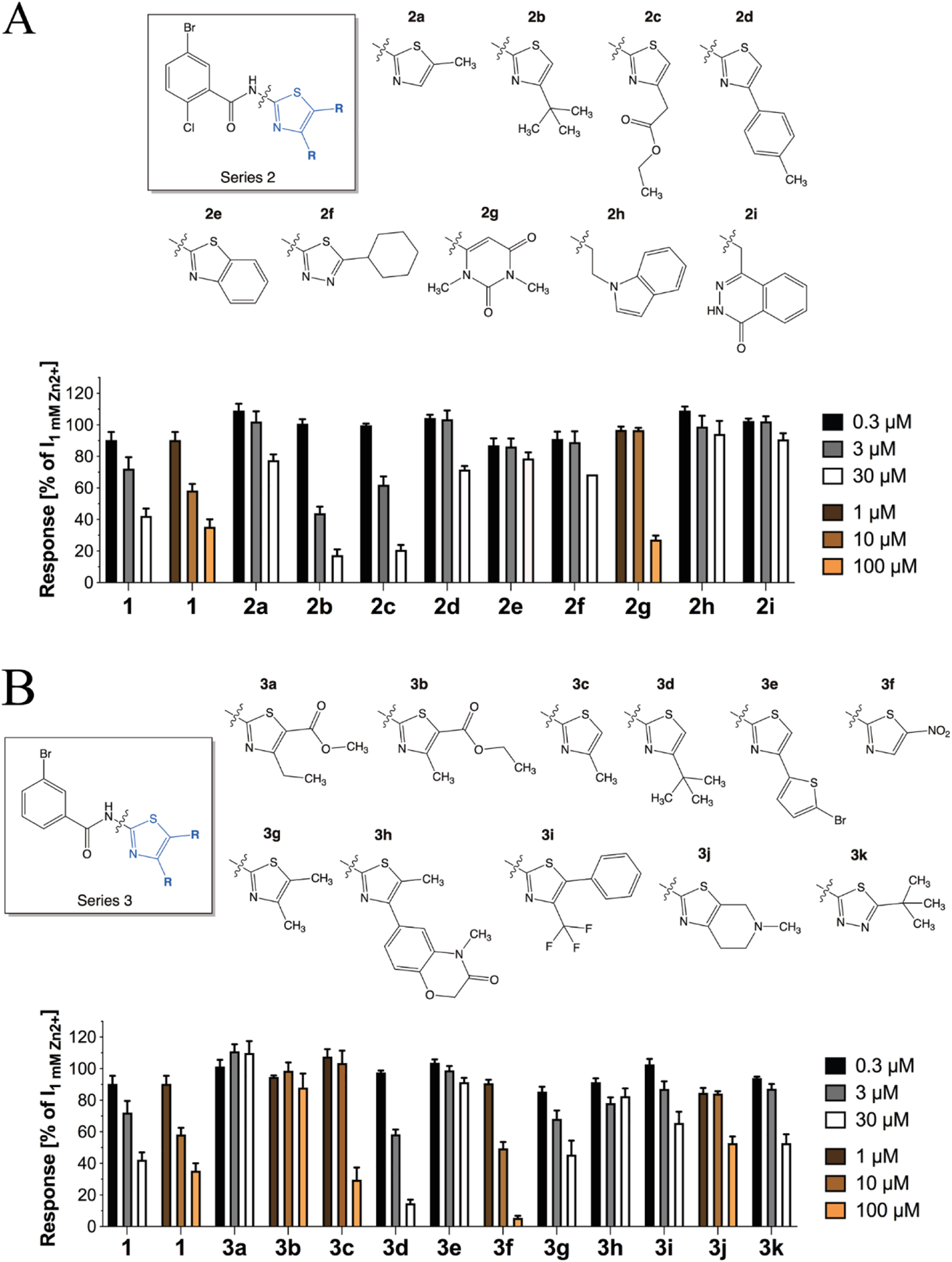
*N*-(thiazol-2-yl)-benzamide analogs with modifications to the thiazole ring. **A**. Chemical structures of *Series 2* analogs (**2a-i**) and the functional properties exhibited by them as antagonists at ZAC in oocytes using 1 mM Zn^2+^ as agonist. Data ate given as mean ± S.E.M. values (n = 4–8). **B**. Chemical structures of *Series 3* analogs (**3a-k**) and the functional properties exhibited by them as antagonists at ZAC in oocytes using 1 mM Zn^2+^ as agonist. Data are given as mean ± S.E.M. values (n = 3–8).

**Fig. 4.**
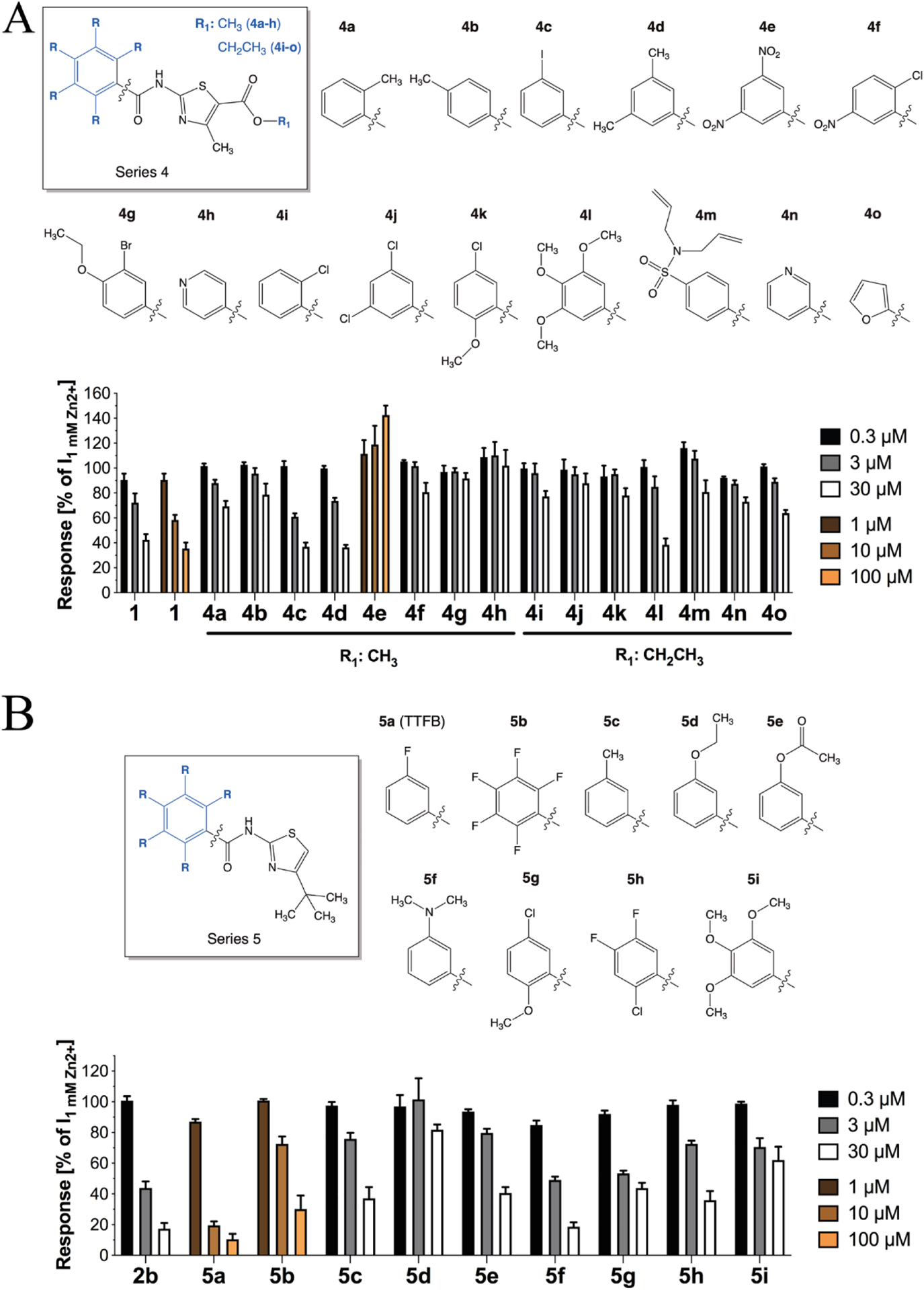
*N*-(thiazol-2-yl)-benzamide analogs with modifications to the phenyl ring. **A**. Chemical structures of *Series 4* analogs (**4a-o**) and the functional properties exhibited by them as antagonists at ZAC in oocytes using 1 mM Zn^+^ as agonist. Data are given as mean ± S.E.M. values (n = 4–8). **B**. Chemical structures of *Series 5* analogs (**5a-i**) and the functional properties exhibited by them as antagonists at ZAC in oocytes using 1 mM Zn^2+^ as agonist. Data are given as mean ± S.E.M. values (n = 4–8).

**Fig. 5.**
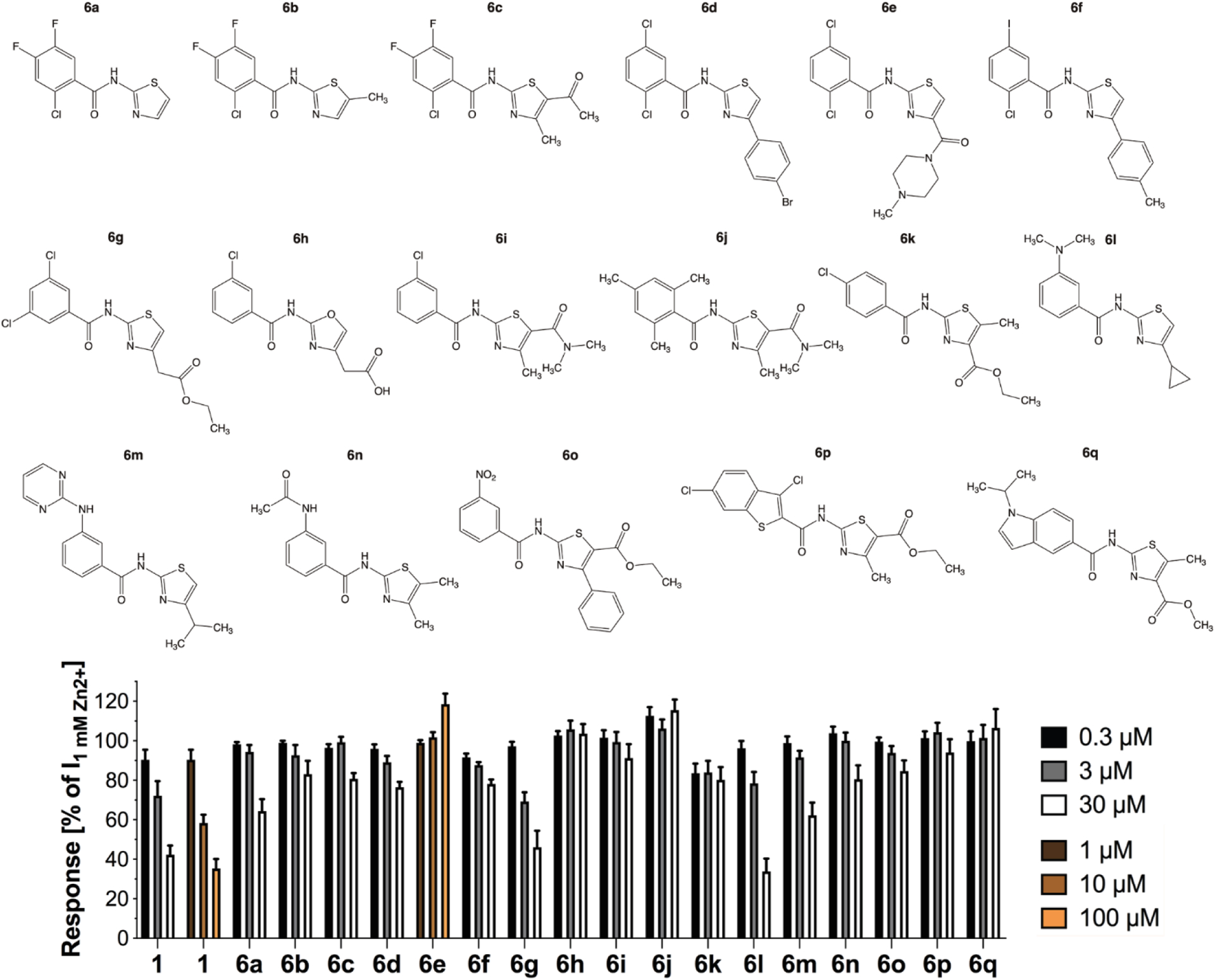
Miscelleneous *N*-(thiazol-2-yl)-benzamide analogs. Chemical structures of *Series* 6 analogs (**6a-q**) and the functional properties exhibited by them as antagonists at ZAC in oocytes using 1 mM Zn^2+^ as agonist. Data are given as mean ± S.E.M. values (n = 5–8).

To elucidate structural determinants for ZAC activity in compound **1** and to search for analogs possessing higher antagonist potency at ZAC compared to this lead, we performed a SAR study in which the functional properties of 61 commercially available *N*-(thiazol-2-yl)-benzamide analogs were characterized at ZAC expressed in oocytes in TEVC recordings. In the following, the SAR results will be presented in the form of five series of analogs (**2a-i, 3a-k, 4a-o, 5a-i, 6a-q**), which all were tested at ZAC at three concentrations (0.3-3-30 μM or 1-10-100 μM, depending on their respective solubility properties) (Figs. 3–5).

#### 3.3.1. Analogs with modifications to the thiazole ring

With *Series 2* and *3*, we probed the importance of the thiazole ring and different substitutions in the 4- and 5-positions of it for the ZAC activity in the *N*-(thiazol-2-yl)-benzamide analog (Fig. 3). Since the nine analogs in *Series 2* all comprise a 5-bromo-2-chlorobenzamido moiety, the functional properties arising from different modifications to or substitutions of the thiazole ring in these analogs can be compared directly to those of **1**. In contrast to the inactivity displayed by the 5-methyl analog **2a** at ZAC, the 4-fert-butyl **(2b)** and 4-ethylacetyl **(2c)** analogs both displayed slightly more potent and efficacious ZAC inhibition than 1, whereas ZAC activity was reduced substantially by the introduction of a 4-(*p*-toIyl) substituent **(2d)** (Fig. 3A). Substitution of the thiazol-2-yl ring for a benzo[(i]thiazol-2-yl **(2e)**, a 5-cyclohexyl-1,3,4-thiadiazole **(2f)** or other bulky ring systems **(2g-i)** also resulted in substantial loss of activity (Fig. 3A).

The 11 analogs in *Series 3* all comprise a 3-bromobenzamido moiety, and thus differences in the functional properties exhibited by the analogs within this series are also directly attributable to their respective thiazole ring substituents. Since we were unable to obtain 2-(3-bromobenzamido)-4-methylthiazole-5-methyl ester from commercial sources, the functional consequences of elimination of the *o*-chloro substituent on the phenyl ring could not be assessed in a direct 1:1 comparison with **1**. However, the fact that **2b** and **3d**, the corresponding 4-(*tert*-butyl)-thiazol-2-yl analogs from *Series 2* and *3*, exhibited comparable antagonist potencies suggested that the *o*-chloro substituent in the phenyl ring is not a key determinant of ZAC activity (Fig. 3A and 3B). Interestingly, very small changes to the 4-methyl and 5-methyl ester substituents on the thiazole ring of **1** yielded the inactive analogs **3a** (4-ethyl, 5-methyl ester) and **3b** (4-methyl, 5-ethyl ester) (Fig. 3B). The 4-methyl analog **3c** also displayed weak ZAC activity, whereas introduction of a *tert*-butyl group in this position yielded a fairly potent analog **(3d)**. Introduction of the bulky 5-bromothiophen-2-yl ring in this position eliminated ZAC activity completely **(3e)**. The 5-nitro analog **(3f)** displayed an IC_50_ ~10 μM, whereas the 4,5-dimethyl analog **3g** was a considerably weaker antagonist. Introduction of bulky aromatic/heteroaromatic substituents in the 4- and/or the 5-position of the thiazole ring essentially eliminated ZAC activity in the analogs **(3h-j)**, and so did the substitution of thiazole for a 5-(*tert*-butyl)-l,3,4-thiadiazole ring **(3k)** (Fig. 3B).

#### 3.3.2. Analogs with modifications to the phenyl ring

With the analogs in *Series 4* and *5*, we investigated the importance of the phenyl ring and the substitution pattern on it for the ZAC activity of the *N*-(thiazol-2-yl)-benzamide analog (Fig. 4). Because of the limited commercial availability of analogs in which the rest of the compound **1** structure was completely conserved, *Series 4* contained both analogs comprising 5-methyl ester **(4a-h)** and 5-ethyl ester **(4i-o)** groups on the thiazole ring (Fig. 4A), and this difference should be kept in mind for SAR comparisons across this analog series. As for the 5-methyl ester analogs **(4a-h)**, the *o*-tolyl **(4a)** and *p*-tolyl **(4b)** analogs were essentially inactive, whereas substitution of the 5-bromo-2-chlorophenyl ring in 1 with 3-iodophenyl **(4c)** or 3,5-dimethylphenyl **(4d)** rings did not alter its antagonist activity substantially (Fig. 4A). In contrast to **4d**, the 3,5-dini-trophenyl analog **(4e)** was completely devoid of antagonist activity at ZAC, and the fact that substitution of the 3-bromo substituent in **1** for a nitro group yielded an inactive analog **(4f)** further substantiated that a *m*-nitro group is unfavourable for ZAC activity. Finally, the introduction of a *p*-ethoxy group or substitution of the phenyl ring for a 4-pyridine resulted in inactive analogs **(4g,h)**. The 5-ethyl ester analogs **(4i-o)** were considerably less informative than the 5-methyl ester analogs, which in light of the inactivity of analog **3b** was not particular surprising. Thus, the inactivity of six of these seven analogs could potentially be rooted in this 5-ethyl ester substituent on the thiazole ring. The *o*-chlorophenyl **(4i)** and 3,5-dichlorophenyl **(4j)** analogs and analog **4k** comprising 5-chloro and 2-methoxy groups in the same positions of the phenyl ring as the 5-bromo and 2-chloro substituents in **1**, respectively, were all inactive. The 3,4,5-trimethoxyphenyl analog **(4l)** displayed weak antagonist activity. Not surprisingly, the analog comprising a big *N, N*-diallylsulfamoyl group in the para-position of the phenyl ring (**4m**) was inactive, as were the 3-pyridin and 2-furan analogs (**4n,o**) (Fig. 4A).

The more potent and notable higher degree of ZAC inhibition mediated by **2b** compared to **1** suggested that introduction of a 4-*tert*-butyl group on the thiazole ring was beneficial for the antagonist activity (Fig. 3A). With *Series 5* we continued to probe the impact of the substituent pattern on the phenyl ring in nine analogs that all comprised this 4-(*tert*-butyl)thiazol-2-yl moiety (Fig. 4B). The 3-fluorophenyl analog **5a** (*N*-(4-(*tert*-butyl)thiazol-2-yl)-3-flurobenzamide, TTFB) was roughly equipotent with **2b** and mediated almost complete inhibition of ZAC at 10 μM, whereas the 2,3,4,5,6-pentafluoropentyl analog **5b** was a considerably weaker ZAC antagonist (Fig. 4B). ZAC activity was also substantially decreased by the introduction of methyl (**5c**), ethoxy (**5d**) or acetyl (**5e**) groups in the 3-position of the phenyl ring, whereas the 3-dimethylamino analog (**5f**) was roughly equipotent with **2b** (Fig. 4B). The 5-chloro-2-methoxyphenyl analog 5g displayed somewhat potent but apparent partial inhibition of ZAC. The 2-chloro-4,5-difluorophenyl analog 5h displayed lower potency as ZAC antagonist than **2b**, which was somewhat surprising considering that some of the more potent analogs in this SAR study comprise m-fluoro (**5a**) and o-chloro (**2b, 2c**) substituents on the phenyl group. Finally, the fact that analogs **5i** and **4l** both were essentially inactive suggests that the 3,4,5-trimethoxy substitution pattern on the phenyl ring is detrimental to ZAC activity.

#### 3.3.3. Miscellaneous analogs with multiple modifications

The 17 analogs in *Series 6* all comprised more than one modification to the *N*-(thiazol-2-yl)-benzamide scaffold compared to **1**, and most of these analogs were completely inactive at ZAC (Fig. 5). The inactivity of analog **6b** was more or less expected, since the 2-chloro-4,5-difluoro-phenyl ring from the moderately potent analog **5h** here is combined with the 5-methyl-thiazol-2-yl moiety known from **2a** to be detrimental for ZAC activity. Moreover, judging from the inactivity of two other 2-chloro-4,5-difluorophenyl analogs, unsubstituted thiazol-2-yl and 5-acyl-4-methylthiazol-2-yl rings are also not beneficial for ZAC activity in the *N*-(thiazol-2-yl)-benzamide (**6a** and **6c**). In light of the inactivity of analogs **2d** and **3e** the inactivity of analogs **6d-f** that also comprise bulky substituents in the 4-position of the thiazole ring was also not particularly surprising. Since **6g** comprises the same 4-ethylacetyl-substituted thiazol-2-yl ring as the potent **2c** analog, the inactivity of this analog must be ascribed to its 3,5-dichlorophenyl moiety. The inactivity of analogs **6h-j** could both arise from the 3-chloro or 2,4,6-tri-methyl substitutions at the phenyl ring or from the 4-acetate and the 5-carboxanride thiazole ring substients. Substitution of the 4-ferf-butyl group at the thiazole in **5f** for a cyclopropyl ring (**6l**) resulted in decreased antagonist potency at ZAC, and introduction of a more bulky 3-substituent in the phenylring further reduced ZAC activity (**6m**) (Fig. 5). Finally, given the weak antagonist activity displayed by other analogs comprising 5-ethyl ester, 4-methyl/5-methyl, 4-methyl or aromatic 4-substituents substituents at the thiazole ring (**2d, 3b, 3c, 3g**), the inactivity of analogs **6n-p** was not surprising.

#### 3.3.4. Detailed functional characterization of selected analogs

**Fig. 6.**
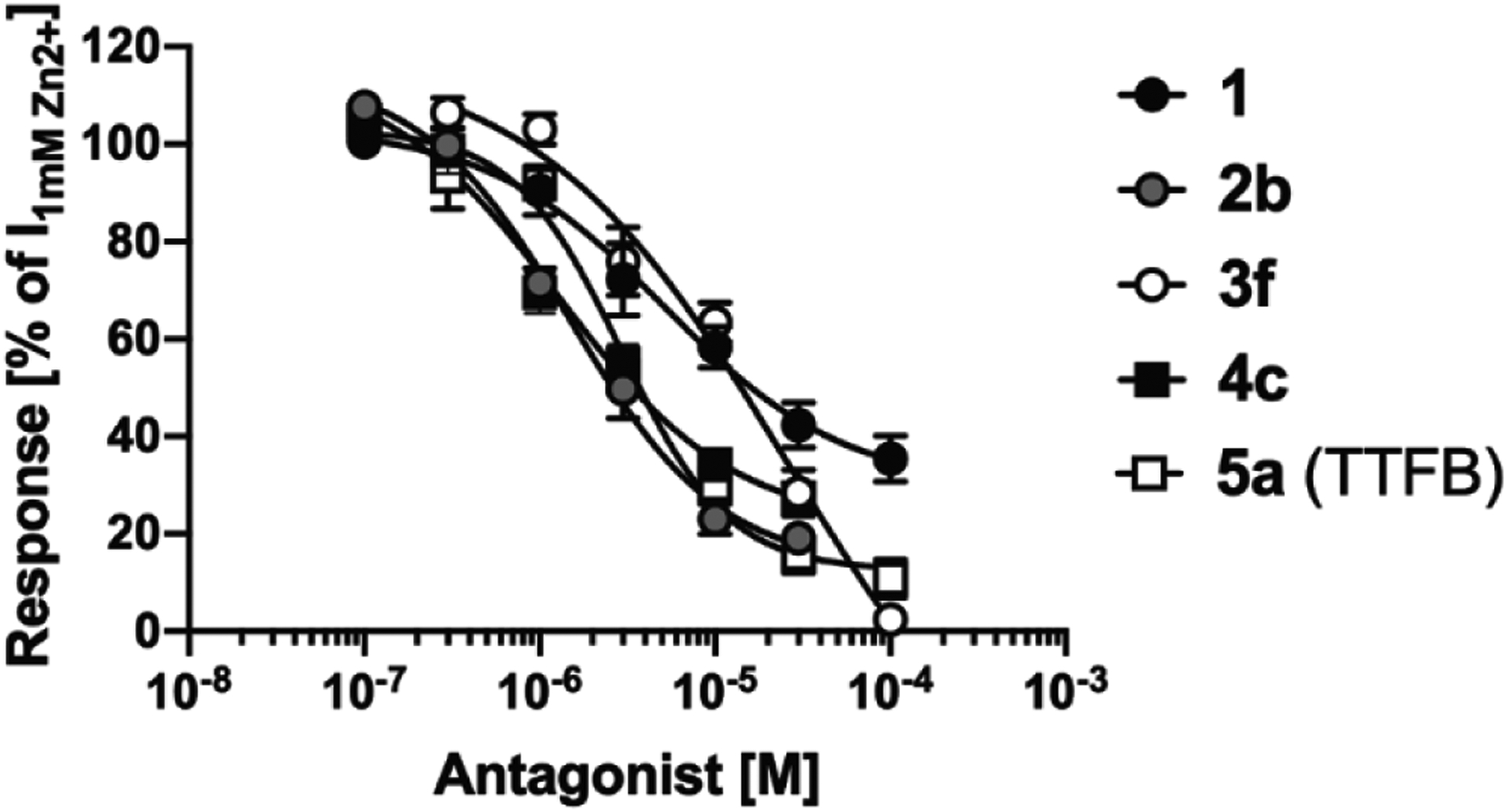
Antagonist properties exhibited by five *N*-(thiazol-2-yl)-benzamide analogs at ZAC expressed in Xenopus oocytes in TEVC recordings. Averaged concentration-inhibition curves for analogs **1**, **2b**, **3f**, **4c** and TTFB (**5a**) at Zn^2+^ (1 mM)-induced currents in ZAC-expressing oocytes. Data are given as mean ± S.E.M. values (n = 5–8). Averaged IC_50_, pIC_50_, n_H_, range of inhibition and n values for the five analogs are given in Table 1.

Following the SAR study, the functional properties of four of the *N-*(thiazol-2-yl)-benzamide analogs were characterized in further detail at ZAC (Fig. 6, Table 1). **2b, 4c** and **5a** (TTFB) were equipotent as ZAC antagonists (IC_50_ values of 1–3 μM), with **1** and **3f** being weaker antagonists. Also notably, and in agreement with observations in the SAR study, **2b, 4c** and **5a** (TTFB) displayed substantially more efficacious ZAC antagonism than **1**, inhibiting the Zn^2+^-induced responses through the receptor completely (Fig. 6, Table 1).

### 3.4. Functional properties and mode of action of TTFB (**5a**) as a ZAC antagonist

The functional properties and mode of action of this novel class of ZAC antagonists were next characterized in further detail using TTFB (**5a**) as representative.

#### 3.4.1. TTFB (**5a**) mediates equipotent and voltage-independent inhibition of agonist-evoked and spontaneous ZAC signaling

**Fig. 7.**
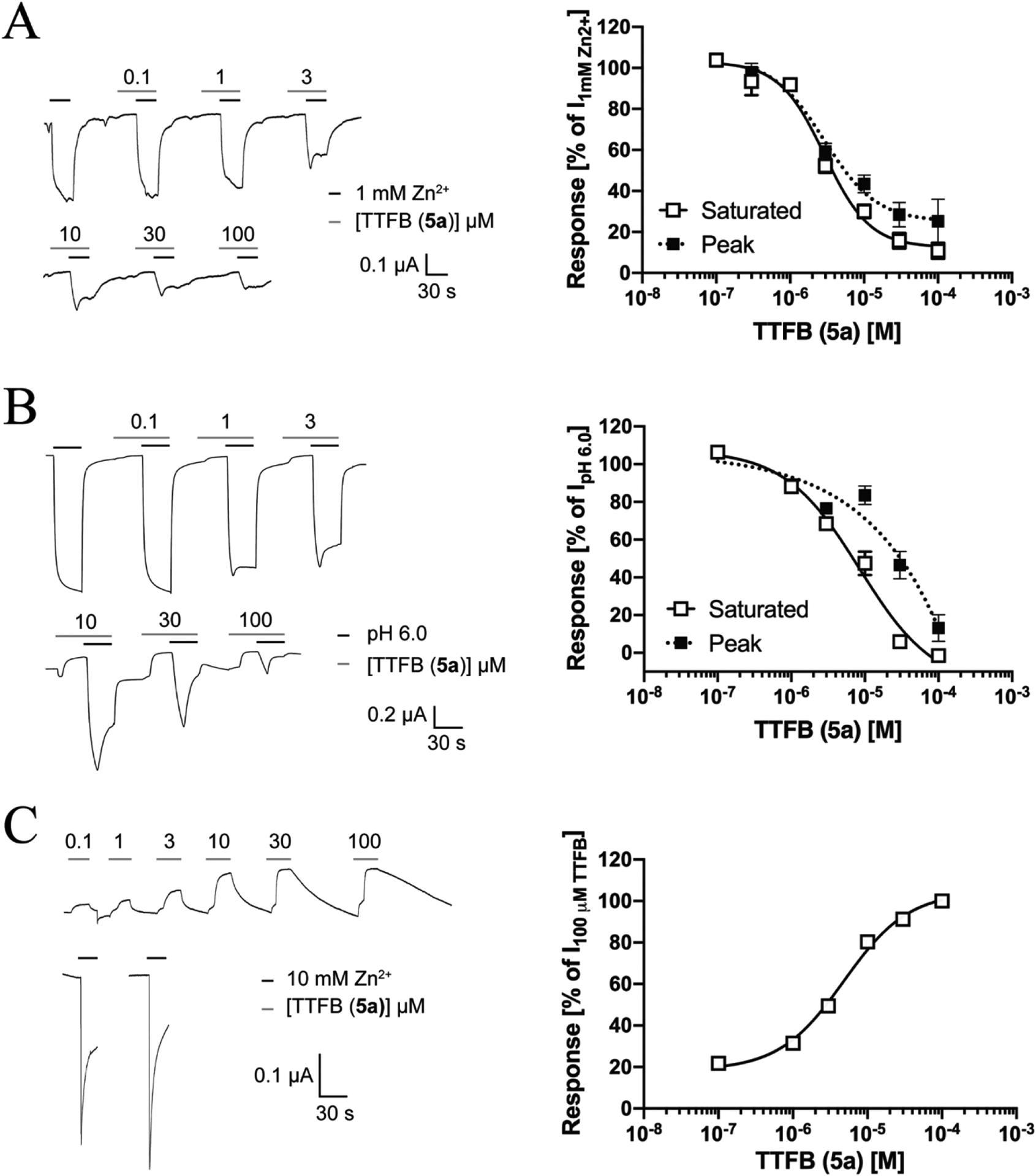
Antagonist properties displayed by TTFB (**5a**) at ZAC expressed in Xenopus oocytes in TEVC recordings. **A**. TTFB inhibits Zn^2+^ -evoked currents through ZAC in oocytes. Representative traces of Zn^2+^ (1 mM)-evoked currents in ZAC-expressing oocytes in the absence and in the presence of increasing concentrations of TTFB (*left*), and averaged concentration-inhibition relationship for TTFB (extracted from recorded saturated and peak responses) at the Zn^2+^ (1 mM)-induced currents in the oocytes (*right*). Data are given as mean ± S.E.M. values (n = 4–7). **B**. TTFB inhibits H^+^ -evoked currents through ZAC in oocytes. Representative traces of H^+^ (pH 6.0)-evoked currents in ZAC-expressing oocytes in the absence and in the presence of increasing concentrations of TTFB (*left*), and averaged concentration-inhibition relationship for TTFB (extracted from recorded saturated and peak responses) at the H^+^ (pH 6.0)-induced currents in the oocytes (*right*). Data are given as mean ± S. E.M. values (n = 4–5). C. TTFB inhibits the constitutive activity exhibited by ZAC in oocytes. Representative traces of the effects of increasing concentrations of TTFB on the leak current in ZAC-expressing oocytes (*left*), and averaged concentration-inhibition relationship for TTFB at the spontaneous currents of ZAC in the oocytes (*right*). Data are given as mean ± S.E.M. values (n = 7).

The antagonist properties exhibited by TTFB at ZAC using Zn^2+^ and H^+^ as agonists and at the spontaneous ZAC activity in oocytes are given in Fig. 7. TTFB inhibited the ZAC responses induced by both Zn^2+^ and H^+^ in a concentration-dependent manner (Fig. 7A–B). Interestingly, TTFB-mediated ZAC inhibition was characterized by a slow on-set, as the peak current initially produced by the agonist in the presence of TTFB was reduced to a lower plateau (the steady-state current) later during the 30 s of the agonist/TTFB co-application (Fig. 7A, *left* and 7B, *left*). Although this slow on-set of inhibition was observed both when using Zn^2+^ and H^+^ as agonists, the degree of it was particularly pronounced in the case of H^+^, which was reflected in the differences observed between the TTFB concentrations-inhibition relationships extracted from recorded peak and steady-state current amplitudes for the two agonists (Fig. 7A, *right* and 7B, *right)*. Extraction of IC_50_ (PIC_50_ ± S.E.M.) values (from the steady-state currents) showed that TTFB displayed slightly higher antagonist potency at Zn^2+^-evoked than at H^+^-evoked ZAC signalling (3.0 μM, 5.52 ± 0.04, n = 7 and 8.5 μM, 5.07 ± 0.10, n = 6, respectively) (Fig. 7A and 7B). The antagonist potency exhibited by TTFB at the spontaneous ZAC activity in the oocytes was comparable to that at the Zn^2+^-evoked response (4.7 μM, 5.32 ± 0.04, n = 7) (Fig. 7C).

**Fig. 8.**
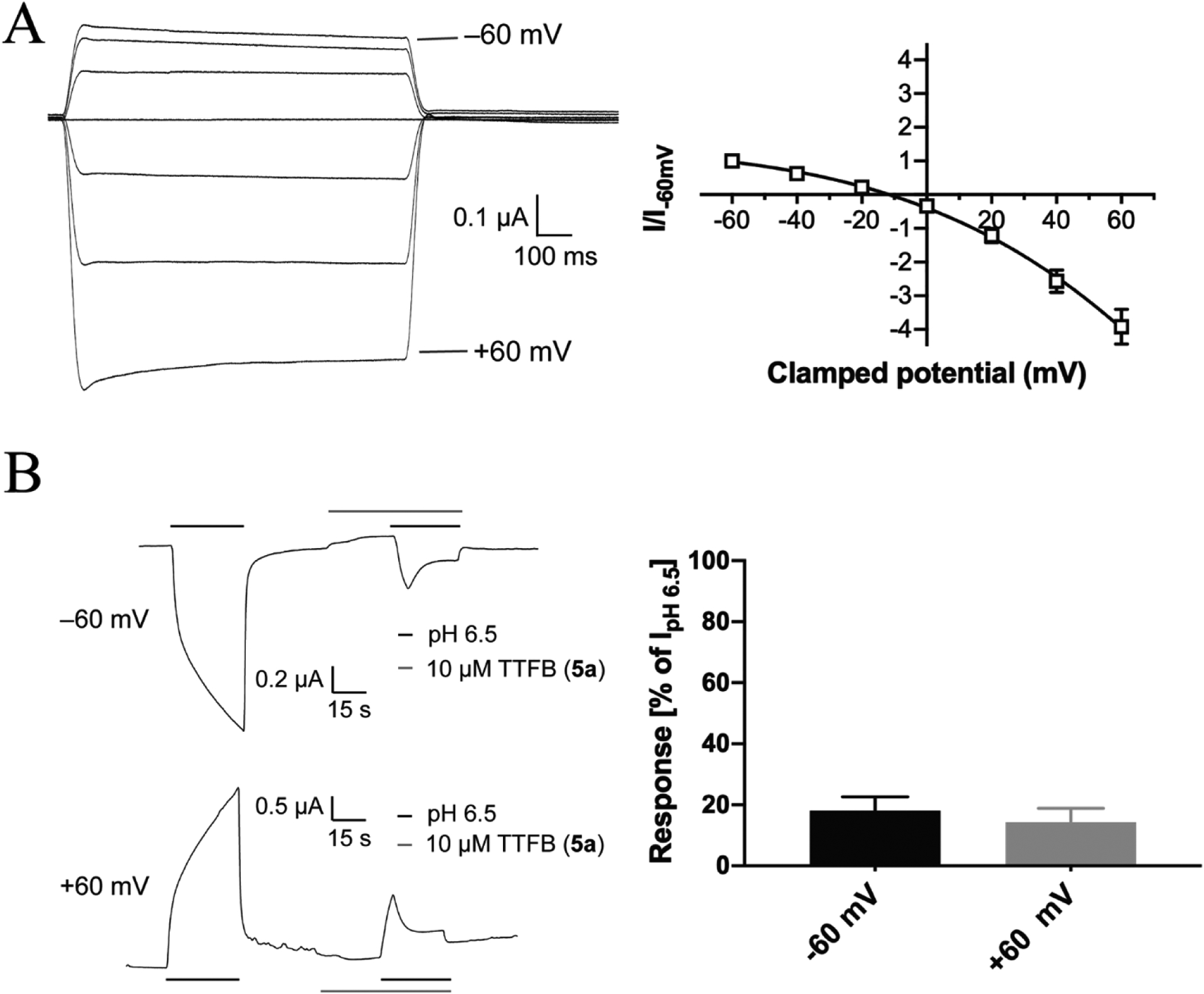
Current-voltage relationship of TTFB (**5a**)-induced currents and its voltage-independent inhibition of H^+^-evoked ZAC currents in Xenopus oocytes in TEVC recordings. **A**. Current-voltage (I-V) relationship of TTFB-mediated inhibition of the spontaneous ZAC currents. Representative traces for currents evoked by applications of TTFB (100 μM) at different holding potentials (*left*) and the averaged IV-relationship displayed by TTFB (100 μM) at ZAC (*right*). Data given as leak-subtracted average current amplitudes normalized to the amplitude of currents recorded at −60 mV (mean ± S.E.M., n = 7). The IV curve was fitted with a third order polynomial model. **B**. Voltage-dependency of the ZAC inhibition mediated by TTFB. Representative traces of currents evoked by H^+^ (pH 6.5) in the absence and presence of TTFB (10 μM) under voltage-clamp of −60 mV and + 60 mV in the same ZAC-expressing oocyte (*left*) and averaged data for the TTFB (10 μM)-mediated inhibition of H^+^ (pH 6.5)-evoked currents in ZAC-expressing oocytes (mean ± S.E.M., n = 4) (*right*).

We also investigated the current–voltage (I-V) relationship of TTFB-mediated inhibition of spontaneous ZAC activity and assessed whether its inhibition of agonist-induced ZAC currents was voltage-dependent (Fig. 8). Analogously to the previously reported I-V-relationship for TC-mediated inhibition of spontaneous ZAC activity [21,25] the TTFB (100 μM)-induced currents recorded from ZAC-oocytes at holding potentials ranging from −60 mV to + 60 mV were characterized by considerably higher amplitudes at positive than at corresponding negative potentials, demonstrating that the spontaneous ZAC currents are outwardly rectifying, and TTFB exhibited an equilibrium potential of −11.5 mV (Fig. 8A). The ZAC inhibition mediated by TTFB was found to be voltage-independent, as the antagonist (10 μM) exerted comparable degrees of inhibition of H^+^-evoked currents in ZAC-oocytes voltage-clamped at −60 mV and + 60 mV (Fig. 8B). Notably, the slow on-set of TTFB-mediated ZAC inhibition was observed at both potentials (Fig. 8B, *left)*.

#### 3.4.2. TTFB (**5a**) is a selective ZAC antagonist

**Fig. 9.**
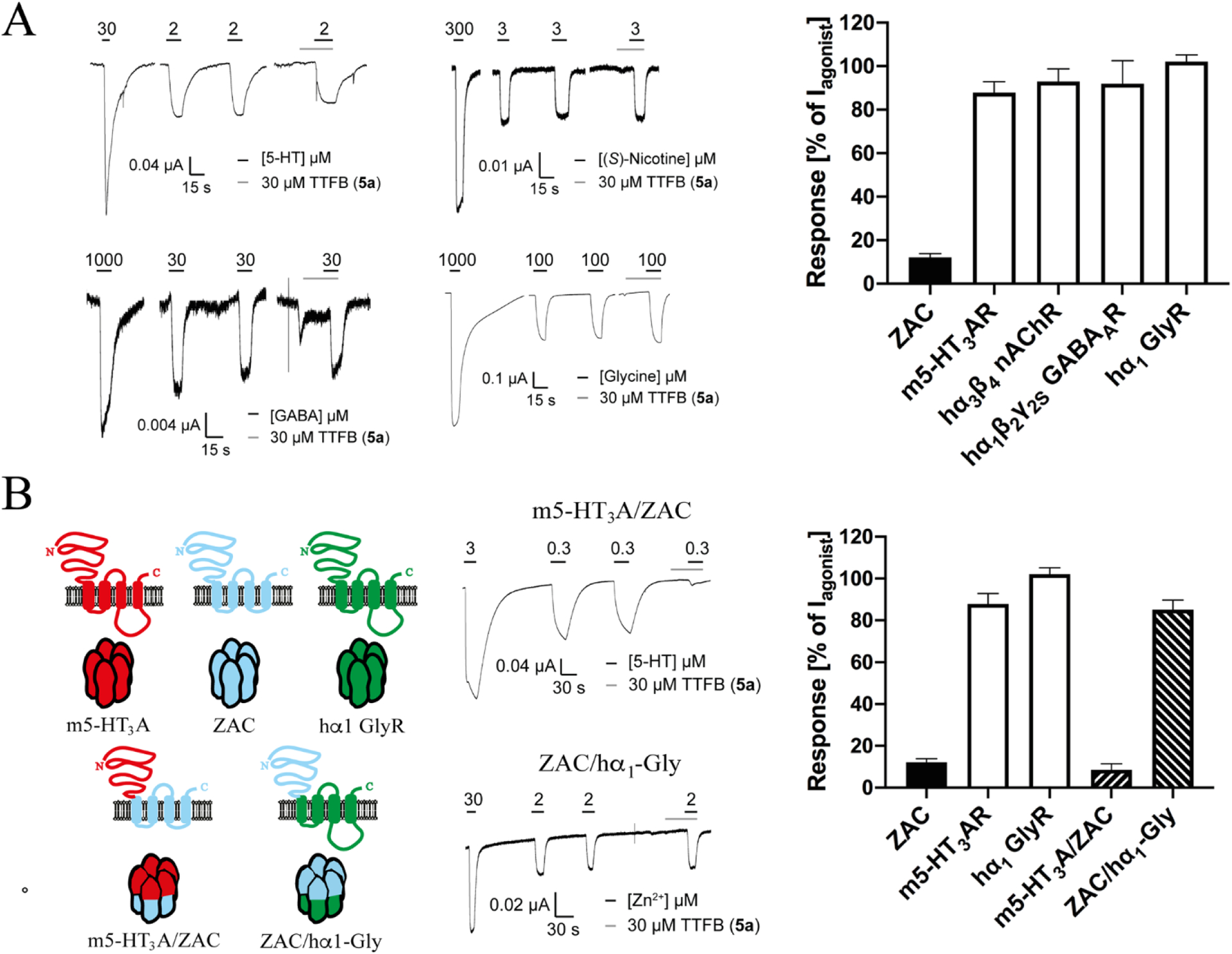
Selectivity profile and mode of action of TTFB (**5a**) as a ZAC antagonist **A**. TTFB is a selective ZAC antagonist. The modulation exerted by TTFB (30 μM) at agonist-induced signalling through ZAC, m5-HT_3_AR, hα_3_β_4_ nAChR, hα_1_β_2_γ_2_s GABA_A_R, and hα_1_ GlyR expressed in oocytes in TEVC recordings. EC_20_–EC_40_ concentrations of the agonists for the respective receptors (1 mM Zn^2+^, 2 μM 5-HT, 3 μM (*S*)-nicotine, 30 μM GABA and 100 μM glycine, respectively) were used for the recordings. Representative traces of agonist-evoked currents in oocytes expressing the respective receptors in the absence and in the presence of 30 μM TTFB (*left*), and averaged data for the currents measured in oocytes expressing the respective receptors in the presence of TTFB (30 μM), normalized to the current evoked by the agonist in the absence of TTFB (I_agonist_) (*right*). The averaged data are given as mean ± S.E.M. values (n = 5–8). **B**. TTFB acts through the transmembrane and/or intracellular domains of ZAC. *Left:* Illustration of the topologies of WT ZAC, WT m5-HT_3_A, WT hα_1_ GlyR, m5-HT_3_A/ZAC and ZAC/hα_1_-Gly subunits and the pentameric complexes assembled from them. *Middle and right:* The modulation exerted by TTFB (30 μM) at the agonist-induced responses through the m5-HT_3_A/ZAC or ZAC/hα_1_-Gly receptors. EC_20_–EC_40_ agonist concentrations for m5-HT_3_A/ZAC or ZAC/hα_1_-Gly (0.3 μM 5-HT and 3 μM Zn^2+^, respectively) were used for the recordings. Representative traces of agonist-evoked currents in oocytes expressing chimeric m5-HT_3_A/ZAC or ZAC/hα_1-_Gly receptors in the absence and in the presence of 30 μM TTFB *(middle)*, and averaged data for the Currents measured in oocytes expressing ZAC, m5-HT_3_AR, hα_1_-Gly, m5-HT_3_A/ZAC and ZAC/hα_1_-Gly in the presence of TTFB (30 μM), normalized to the current recorded in the absence of TTFB (I_agonist_) (*right*). The averaged data for m5-HT_3_A/ZAC and ZAC/hα_1_-Gly are given as mean ± S.E.M. values (n = 6–7), and the averaged data for ZAC, m5-HT_3_AR and hα_1_ GlyR from Fig. 9A are presented for comparison.

The selectivity profile of TTFB as ZAC antagonist was assessed by investigating its functional properties at representatives from each of four classical CLR subfamilies: the m5-HT_3_AR, the hα_3_β_4_ nAChR, the hα_1_β_2_γ_2s_ GABA_A_R and the hα_1_ GlyR (Fig. 9A). In these recordings, 30 μM TTFB was preapplied onto the oocyte for 30 s before TTFB (30 μM) was co-applied with an EC_20_–EC_40_ concentration of the agonist for the specific receptor, thus testing simultaneously for putative activity of TTFB as an agonist, as an antagonist or as a PAM at the receptor. TTFB (30 μM) did not display significant activity at any of these four representative classical CLRs (Fig. 9A).

#### 3.4.3. TTFB (**5a**) acts through the TMD/ICD of ZAC

Functional receptors formed from chimeric subunits fusing the ECD of one CLR with the TMD and ICD of another have been used extensively to study the molecular basis for CLR signaling and to identify receptor domains involved in modulator binding [34–38]. In a companion paper [31] we report that chimeric m5-HT_3_A/ZAC and ZAC/hα_1_-Gly subunits both assemble into functional homomeric receptors when expressed in *Xenopus* oocytes (Fig. 9B, *left)*. In this work, we took advantage of the fact that these two chimeric receptors comprise the ECD or the TMD-ICD of ZAC fused with the complementary domain from another CLR not modulated by TTFB (Fig. 9A) to delineate the ZAC domain targeted by the antagonist. Interestingly, TTFB (30 μM) mediated complete inhibition of the 5-HT-induced response through m5-HT_3_A/ZAC, whereas it displayed negligible effect on the Zn^2+^-induced response through ZAC/hα_1_-Gly (Fig. 9B, *middle* and *right)*. These black-and-white modulatory properties of TTFB (30 μM) exhibited at the two chimeric receptors strongly indicates that the binding site of the modulator resides within the TMD and/or ICD of ZAC.

## 4. Discussion

In the present work we have discovered the first class of selective ZAC antagonists and delineated the functional properties and mode of action of these at the receptor.

### 4.1. Structure-activity relationship (SAR) of the *N*-(thiazol-2-yl)-benzamide analog as a ZAC antagonist

When developing modulators based on a hit from a compound library screening, chances of optimizing the pharmacological properties of the hit as well as the ability to elucidate the SAR in a systematic manner are very dependent on the analogs accessible. These limitations are also evident in the present study, where the SAR was based exclusively on commercially available analogs. Not all 61 analogs of **1** included in the study were informative with regard to the structural determinants for ZAC activity in the *N*-(thiazol-2-yl)-benzamide analog, and often the consequences of a modification in a certain position of this scaffold could not be assessed unambiguously due to differences in other parts of two analogs as well. Nevertheless, we propose that several interesting SAR observations about the *N*-(thiazol-2-yl)-benzamide analog as ZAC antagonist can be extracted from the study.

The thiazole ring system was found to be integral for the ZAC activity of the *N*-(thiazol-2-yl)-benzamide analog, as substitution of this ring for other ring systems (**2f-i, 3k)** or fusion of it with phenyl or cycloalkyl rings (**2e, 3j**) yielded inactive analogs. Introduction of small electron-withdrawing substituents (methoxy, nitro) in the 5-position of the thiazole ring yielded some of the more potent analogs in the series (**1**, **3f**), whereas analogs with electron-donating groups (methyl, phenyl) in this position were inactive (**2a, 3g,i**) (Fig. 6B). The apparent importance of the electronic characteristics of the 5-substituent could arise from a direct impact on its ability to form interactions in the modulator binding site or from an overall effect on the electron distribution in the thiazole ring. In light of the relative potent activity displayed by several 5-methoxy analogs (**1, 4c,d**), the inactivity displayed by all 5-ethoxy (**3b, 4i-o**) and 5-(*N*,*N*-dimethyl)amide (**6i,j**) analogs was surprising (Figs. 3–5). This suggests that the 5-substituent projects towards and potentially forms interactions with residues lining the modulator binding site and, if sufficiently big, will introduce a steric clash with these. Analogs unsubstituted at the 5-position included inactive compounds (**2d, 3e**) as well as both weak (**3c**) and potent ZAC antagonists (**2b,c, 3d**), a range in potency primarily attributable to the identity of the 4-substituent in their respective thiazole rings. Introduction of bulky aliphatic and ester groups in the 4-position (**2c,b, 3d**) increased ZAC activity substantially compared to analogs with a 4-methyl (**3c**), whereas ethyl or aromatic/heteroaromatic groups in this position was much less favourable for ZAC activity (**2d, 3a**,**e**) (Fig. 3).

The benzamide moiety was retained in 59 of the 62 analogs in the SAR study, which thus mainly focused on the importance of the phenyl ring substitution pattern for the ZAC activity of the *N*-(thiazol-2-yl)-benzamide analog, primarily with the **4a-h** and **5a-i** analogs. Introduction of a single *ortho-* or *para*-methyl group in the phenyl ring yielded inactive analogs (**4a,b**). As evidenced by the potent ZAC antagonism exhibited by the 5-bromo-2-chlorobenzamido analogs **1** and **2b,c**, an *ortho*-chloro substituent can be accommodated in the *N*-(thiazol-2-yl)-benzamide without loss of activity, albeit the similar antagonist potencies displayed by **2b** and **3d** also suggest that this substituent does not contribute significantly to binding affinity. The inactivity of **4g** contrasts the potent ZAC activity displayed by **1** and suggests that *para*-substitution in the phenyl ring is unfavorable for modulator binding. Finally, analogs comprising a single methyl, fluoro, iodo or *N*,*N*-dimethylamide group in the *meta*-position possessed potent activity (**4c, 5a,c,f**), whereas the *m*-aceto and *m*-ethoxy analogs **5d,e** were inactive (Fig. 6B). Since ZAC activity thus was retained in analogs with fairly big *meta-substituents*, the contrasting activities exhibited by **4d,e** and **6g** indicate that *m*-substituent bulk could be more critical for the analogs with 3,5-disubstituted phenyl rings and that residues in the modulator binding pocket may present steric clash with one of the two *meta*-substitutions in the **4e** (3,5-dinitro) and **6g** (3,5-dichloro) analogs. Moreover, the fact that most analogs comprising a disubstituted phenyl ring (including **4a, b,e-h, 5d,e,i, 6g**) were inactive raises another point. While no analogs comprising an unsubstituted phenyl group was included in the study, the *m*-fluoro analog TTFB (**5a**) was one of the most potent ZAC antagonists in the series, indicating that substitutions to the phenyl ring in the *N-*(thiazol-2-yl)-benzamide analog in general may not be beneficial for ZAC modulation.

While we obviously could have hoped for analogs with even more increased antagonist potencies at ZAC to come out of the SAR study, the functional properties exhibited by analogs **2b, 4c** and **5a** at ZAC still constitute improvement compared to those of **1** (Fig. 6A). It is also important to stress that derivatization strategies not pursued in this study potentially could form the basis for development of more potent ZAC antagonists. For example, since 4-*tert*-butyl/4-ethylacetyl and 5-nitro/5-methoxy substitutions to the thiazole ring seem to have isolated beneficial effects on ZAC activity in the *N*-(thiazol-2-yl)-benzamide analog (**1, 2b,c, 3c,f**), it would be interesting to probe whether combining these substituents in the *N*-(thiazol-2-yl)-benzamide analog would have additive or synergistic effects on ZAC antagonist potency.

### 4.2. Mode of action of the *N*-(thiazol-2-yl)-benzamide analog as ZAC antagonist

Our investigations into the underlying mechanism for the ZAC inhibition exerted by the *N*-(thiazol-2-yl)-benzamide analogs unequivocally demonstrate that the compounds act as NAMs at ZAC. In light of its pronounced selectivity for ZAC over m5-HT_3_AR and hα_1_ GlyR (Fig. 9A), the robust TTFB-mediated inhibition of m5-HT_3_A/ZAC signalling and its negligible effect on ZAC/hα_1_-Gly signalling pinpoints the *N*-(thiazol-2-yl)-benzamide analog binding site to reside within the TMD-ICD of ZAC (Fig. 9B). Since both Zn^2+^ and H^+^ have been proposed to act through the ECD of ZAC [31], this also concords with the largely non-competitive mode of inhibition displayed by compound **1** on Zn^2+^-induced ZAC signalling (Fig. 2C). The classification of the *N*-(thiazol-2-yl)-benzamide analog as a ZAC NAM could actually be argued to be premature, since it still remains a question whether Zn^2+^, Cu^2+^ and/or H^+^ or another yet unidentified transmitter is the “true” endogenous agonist for the receptor. However, given that the presently identified ZAC agonists act through the ECD and that any other putative “true” ZAC transmitter would have to be the first endogenous CLR agonist not acting through the ECD in order for the *N*-(thiazol-2-yl)-benzamide analog not to be a NAM, we nevertheless consider this a reasonable claim.

While a possible involvement of the ICD in *N*-(thiazol-2-yl)-benzamide binding to ZAC cannot be completely ruled out based on the black-and-white modulation displayed by TTFB at the m5-HT_3_A/ZAC and ZAC/hα_1_-Gly receptors, it seems unlikely (Fig. 9B). Classical CLR signaling is known to be modulated by phosphorylation of ICD residues and by binding of intracellular proteins to this domain [39,40], but to our knowledge no small-molecule CLR modulators have been reported to act through this domain. Moreover, the fact that the second intracellular loop in ZAC is very short (~40 residues) compared to the corresponding loops in the classical CLRs makes a putative involvement of this domain in TTFB binding even more unlikely. In contrast, a considerable amount of experimental data from structural biology, biophysical and mutagenesis studies over the years have demonstrated that the CLR TMD comprises several allosteric sites, and a multitude of modulators have been proposed to act through intra-subunit or inter-subunit sites, through sites formed by TMD regions and their surrounding lipid bilayer, through ECD/TMD interface sites or as regular ion channel blockers [12,28,41–47]. The insights into the molecular architecture of these allosteric sites could guide the search for the *N*-(thiazol-2-yl)-benzamide binding site in the ZAC TMD in future studies.

The interesting slow on-set of the TTFB-mediated ZAC inhibition can not be ascribed to overall slow on-binding of the antagonist to the receptor. Whereas TTFB seems unable to establish binding equilibrium with ZAC during the 30 s-preincubation step, the subsequent co-application of it with agonist clearly facilitates its receptor binding (Fig. 7A, 7B and 8B), and analogously, TTFB-mediated inhibition of spontaneous ZAC activity occurs immediately after ligand application and reaches a steady-state well within 30 s (Fig. 7C). Thus, we propose that the observed slow on-set of ZAC inhibition could be a reflection of state-dependent antagonism, where TTFB preferentially targets the active conformation (be it the constitutively active or the agonist-bound/open channel) over the resting conformation of ZAC. State-dependency in ion channel antagonists is often associated with channel blockers acting through sites in the ion pore [48–50] but it can also arise from other mechanisms [51], and thus this observation does not further elucidate the location of the *N*-(thiazol-2-yl)-benzamide binding site in the ZAC TMD. However, the substantially larger difference between peak and steady-state currents observed for TTFB-mediated inhibition of H^+^-evoked currents than for its inhibition of Zn^2+^-evoked currents is interesting, as it could have both molecular and kinetic origins. In the molecular scenario, the state-dependency of the TTFB-mediated inhibition is agonist-specific, meaning that the structural composition of the TTFB binding site, and with that the ability of the antagonist to bind to it, differs in the Zn^2+^- and H^+^-stabilized active ZAC conformations. While TTFB thus may exhibit little preference for the Zn^2+^-bound active conformation over the resting ZAC conformation, resulting in a modest difference between peak and steady-state currents (Fig. 7A), TTFB binding affinity to the proton-activated ZAC could be vastly higher than its binding affinity to the resting conformation, reflected in the big difference between the two currents for this agonist (Fig. 7B and 8B). In the kinetic scenario, TTFB displays similar preference for the Zn^2+^- and H^+^-bound active ZAC conformation over the resting conformation, and thus the different peak/steady-state current ratios observed for the two agonists are rooted in their different kinetic properties. H^+^-evoked currents through ZAC have been shown both to activate and decay considerably faster than those evoked by Zn^2+^ [22], and given the negligible desensitization of ZAC and the presumed limited contribution of deactivation in the continuous presence of agonist during the recordings, peak current amplitudes evoked in the presence of TTFB will mainly be determined by the activation kinetics of the agonist and how these compare to the kinetic properties of TTFB binding to the active ZAC conformation. Thus, at equi-effective Zn^2+^ and H^+^ concentrations, the faster activator of the two agonists (H^+^) will induce a higher maximal number of open channels during the initial phase of the agonist/TTFB co-application than the slower agonist (Zn^+^), and the change in the current arising from the subsequent establishment of the kinetic equilibrium between the agonist and TTFB (steady-state) will inevitably be bigger for the agonist having the bigger kinetic edge as there will be a greater accumulation of agonist-bound, blocked channels.

### 4.3. The *N*-(thiazol-2-yl)-benzamide analog as a pharmacological tool for ZAC

While TTFB and the other active N-(thiazol-2-yl)-benzamide analogs in this series represent the first selective ZAC ligands published to date, some considerations should be given to their applicability as pharmacological tools.

**Fig. 10.**
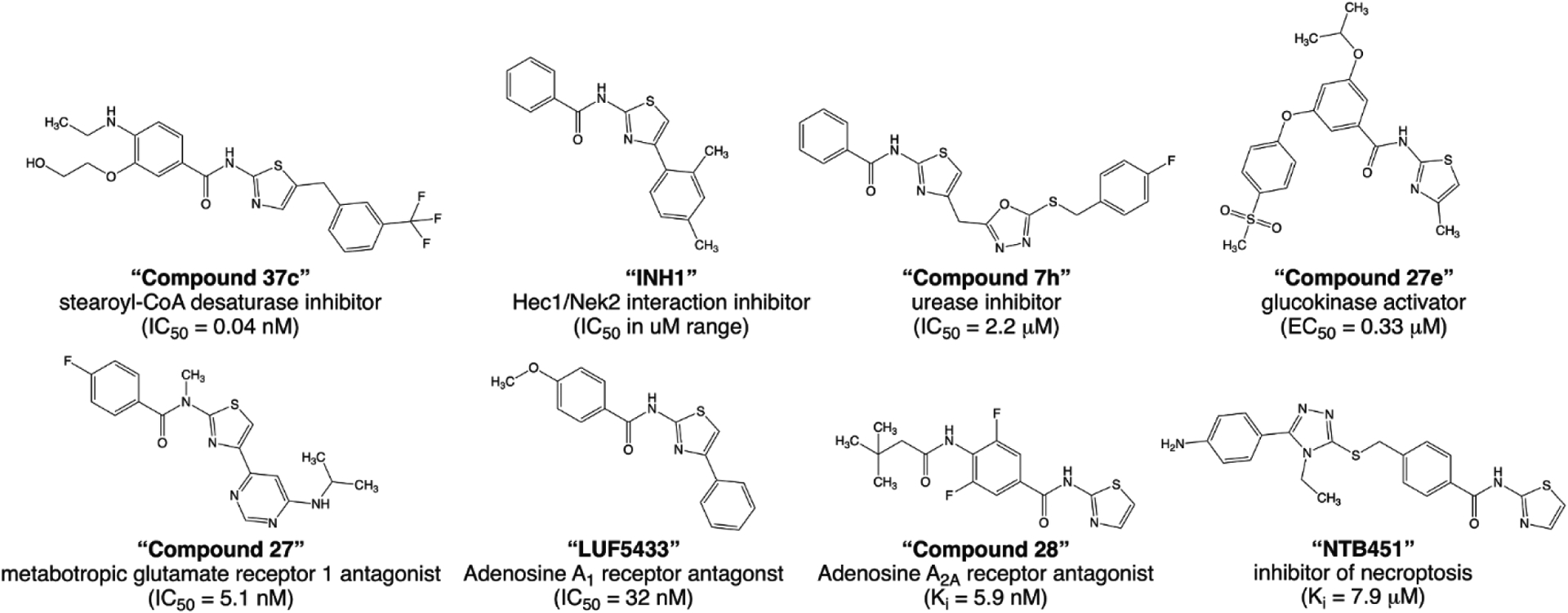
Examples of previously published ligands comprising the *N*-(thiazol-2-yl)-benzamide moiety. The chemical structures, main targets and pharmacological activities reported for eight different ligands comprising the *N*-(thiazol-2-yl)-benzamide moiety [52–59].

Although we only have probed the putative off-target effects of TTFB by testing it at one representative from each of the four classical CLR subfamilies, we propose that the pronounced selectivity exhibited by the antagonist for ZAC over m5-HT_3_AR, hα_3_β_4_ nAChR, hα_1_β_2_γ_2S_ GABA_A_R and hα_1_ GlyR is likely to extend to the other members of the CLR superfamily (Fig. 9A). Considering the very low homology (<20% amino acid sequence identity) between ZAC and these CLRs [21], it is perhaps not surprising that it is possible to identify selective ZAC modulators. However, whether TTFB and its analogs overall are selective for ZAC over other off-targets is an entirely different matter. The *N*-(thiazol-2-yl)-benzamide scaffold has been used extensively in medicinal chemistry development of ligands for a diverse range of targets (exemplified in Fig. 10) [52–59]. Judging from the ZAC SAR determined in this study, all of these previously published compounds comprise additional moieties and/or substitutions at the *N*-(thiazol-2-yl)-benzamide scaffold that most likely would render them inactive at ZAC. Conversely, it can not be ruled out that the ZAC antagonists identified in this study could hold activity at some of these other targets, and a ligand displaying a functional potency of ~1 μM at its target can not be automatically assumed not to possess off-target activities in relevant concentration ranges.

Another question is whether TTFB and the other *N*-(thiazol-2-yl)-benzamide analogs presented here potentially could be applied as a pharmacological tool for studies of ZAC *in vivo*. We have not investigated the pharmacokinetic properties of TTFB, but given its moderate target potency and its limited solubility it seems unlikely that systemic administration of the drug would produce sufficiently high concentrations of the free (unbound) compound to achieve significant ZAC inhibition *in vivo*. Moreover, the absence of an orthologous *ZACN* gene in rat and mouse genomes also rules out that such studies can be performed in the species most often used for *in vivo* investigations. Instead, TTFB and its analogs could be useful tools in focused electrophysiological or other functional *in vitro/ex vivo* studies of native ZAC signalling by use of tissue or cell lines derived from human or other mammalian species.

## 5. Conclusion

In conclusion, TTFB and the other active *N*-(thiazol-2-yl)-benzamide analogs constitute the first class of selective antagonists of ZAC, and we propose that the antagonists represent a valuable addition to the limited pharmacological tool box presently available for ZAC and could aid future investigations into this enigmatic member of the CLR superfamily. The *N*-(thiazol-2-yl)-benzamide analogs were found to act as NAMs through a binding site in its TMD and to mediate state-dependent inhibition of ZAC, which further substantiates the complexity of CLR signalling and of the ligand-mediated modulation of them through allosteric sites.

## Figures and Tables

**Table 1:** Functional properties exhibited by five *N*-(thiazol-2-yl)-benzamide analogs at ZAC expressed in oocytes using Zn^2+^ (1 mM) as agonist determined by TEVC electro-physiology. IC_50_ (in μM), pIC_50_ ± S.E.M., Hill coefficient (n_H_ ± S.E.M.) and the fitted inhibition range (in %) are given, and the number of experiments for the data are indicated (n). The averaged concentration-inhibition relationships for the five analogs at ZAC are presented in Fig. 6.

	IC_50_ (μM)	pIC_50_ ± S.E.M.	n_H_ ± S.E.M.	Inhibition (%)	n
**1**	4.1	5.39 ± 0.17	−1.3 ± 0.13	69 ± 3	6
**2b**	1.3	5.88 ± 0.05	−1.2 ± 0.12	88 ± 3	7
**3f** ^a^	~20	~4.7	n.d.	n.d.	4
**4c**	1.0	5.99 ± 0.08	−0.89 ± 0.06	78 ± 3	5–7
**5a** (TTFB)	3.0	5.52 ± 0.04	−1.5 ± 0.08	91 ± 3	6

n.d., not determinable.

a The concentration-inhibition curve for this analog was not completed within the tested concentration range.

The IC_50_ and pIC_50_ values for the analog are estimated under the assumption that it would mediate complete inhibition.

## Abbreviations:

ACh: acetylcholine
5-HT: 5-hydioxytiyptaniine
5-HT_3_R: 5-HT_3_ receptor
CLR: Cys-loop receptor
EAAT3: excitatory amino acid transporter subtype 3
ECD: extracellular domain
FMP: FLIPR Membrane Potential Red
GABA_A_R: GABA_A_ receptor
GlyR: glycine receptor
ICD: intracellular domain
nAChR: nicotinic acetylcholine receptor
NAM: negative allosteric modulator
PAM: positive allosteric modulator
SAR: structure-activity relationship
TC: tubocurarine
TEVC: two-electrode voltage clamp
TMD: transmembrane domain
TTFB: *N*-(4-(*tert*-butyl)thiazol-2-yl)-3-fluorobenzamide
WT: wild type
ZAC: Zinc-Activated Channel
